# Investigation of Antioxidant and Antimicrobial Properties of Sunda Porcupine’s (*Hystrix javanica*, F.Cuvier, 1823) Quills Ethanolic Crude Extract

**DOI:** 10.21315/tlsr2024.35.3.1

**Published:** 2024-10-07

**Authors:** Muhamad Arif Budiman, Pamungkas Rizki Ferdian, Tri Hadi Handayani, Rizki Rabeca Elfirta, Masrukhin, Herjuno Ari Nugroho, Ni Luh Putu Rischa Phadmachanty, Wartika Rosa Farida, Ardya Widyastuti, Dianita Dwi Sugiartanti

**Affiliations:** 1 Faculty of Medicine, Universitas Muhammadiyah Prof. DR. HAMKA, Tangerang City, Banten 15153, Indonesia; 2 Research Centre for Applied Zoology, National Research and Innovation Agency (BRIN), Bogor, West Java 16911, Indonesia; 3 Research Centre for Applied Microbiology, National Research and Innovation Agency (BRIN), Bogor, West Java 16911, Indonesia; 4 Research Centre for Biosystematic and Evolution, National Research and Innovation Agency (BRIN), Bogor, West Java 16911, Indonesia; 5 DialoVet Animal Care, Bogor, West Java 16913, Indonesia; 6 Indonesian Institute for Testing Standard Instrument of Veterinary, Bogor, West Java 16124, Indonesia

**Keywords:** Antioxidant, Antimicrobial, GCMS, Sunda Porcupine, *Hystrix javanica*

## Abstract

The Sunda porcupine (*Hystrix javanica*, F.Cuvier, 1823) is a rodent-mammal species native to Indonesia and is utilised in traditional medicine for the treatment of various ailments. Some ethnic communities in Indonesia have traditional beliefs regarding Sunda porcupine’s quills, which are thought to relieve back pain and toothache. Despite this traditional knowledge, there is limited scientific research on the topic. The aim of this study was to identify active compound in an ethanolic crude extract of Sunda porcupine’s quills, and to evaluate its antioxidant and antimicrobial properties. The antioxidant activity was evaluated using 1,1-diphenyl-2-picrylhydrazyl (DPPH)-free radical scavenging assay while the antimicrobial activity was evaluated through microdilution resazurin assay. The total phenolic and flavonoid contents were also determined to support the antioxidant properties. The active compounds were identified using gas chromatography-mass spectrophotometer (GCMS) with the National Institute of Standards and Technology (NIST-11) library. The result showed that the extract possesses antioxidant properties (IC_50_ 138.93 μg/mL) and antimicrobial properties against *Escherichia coli* (*E. coli*), *Staphylococcus aureus* (*S. aureus*), *Bacillus subtilis* (*B. subtilis*), *Pseudomonas aeruginosa* (*P. aeruginosa*) and *Candida albicans* (*C*. *albicans*) (IC_50_ range 0.40 mg/mL–33.05 mg/mL). Total phenolic content (TPC) and total flavonoid content (TFC) were 27.29 ± 2.20 mgGAE/g and 27.09 ± 1.66 mgQE/g, respectively. A total of 24 active compounds from the crude extract were identified. As much as five compounds serve as antioxidant agents, including: butylated hydroxytoluene; eicosane; 1-iodo-hexadecane; methyl ester hexadecanoic acid; and L-(+)-ascorbic acid 2,6-dihexadecanoate. Furthermore, as much as 11 compounds serve as antimicrobial agents, including: tetradecane; pentadecane; 2-isopropyl-5-methyl-1-heptanol; hexadecane; butylated hydroxytoluene; eicosane; 1-iodo-hexadecane; methyl ester hexadecanoic acid; benzenepropanoic acid, 3,5-bis(1,1-dimethylethyl)-4-hydroxy-, methyl ester; L-(+)-ascorbic acid 2,6-dihexadecanoate; and octadecanoic acid. This study provides scientific validation for the use of the Sunda porcupine’s quills in traditional medicine and highlights the potential for further research in animal bioprospecting.

**Highlights**
- The extract possesses antioxidant properties (DPPH IC50 138.93 μg/mL) and antimicrobial properties against *E. coli*, *P aeruginosa*, *S. aureus*, *B. subtilis* and *C. albicans* (IC50 range 0.40 mg/mL–33.05 mg/mL).
- Total phenolic and total flavonoid content were 27.29 ± 2.20 mgGAE/g and 27.09 ± 1.66 mgQE/g.
- A total of 24 active compounds from the crude extract were identified. As much as 5 compounds serve as antioxidant agents, including: butylated hydroxytoluene; eicosane; 1-iodo-hexadecane; methyl ester hexadecanoic acid; and L-(+)-ascorbic acid 2,6-dihexadecanoate. Furthermore, as much as 11 compounds serve as antimicrobial agents, including: tetradecane; pentadecane; 2-isopropyl-5-methyl-1-heptanol; hexadecane; butylated hydroxytoluene; eicosane; 1-iodo-hexadecane; methyl ester hexadecanoic acid; benzenepropanoic acid, 3,5-bis(1,1-dimethylethyl)-4-hydroxy-, methyl ester; L-(+)-ascorbic acid 2,6-dihexadecanoate; and octadecanoic acid.

## INTRODUCTION

Animal-based medicine has been used for centuries in traditional medicine systems around the world. However, its use is not as widespread as that of plant-based medicine due to various factors such as cultural, religious, conservation and abundance considerations. The medicine can be derived from diverse range of animal materials, including animal metabolites, body parts or non-animal components such as bird nests, bee hives and cocoons. Inspired by ethnobiology and ethnomedicine, research into animal-based medicine has been conducted globally, including in Indonesia. Mardiastuti *et al*. (2021) documented the use of a variety of animal species in Indonesian traditional medicine, including 59 mammal species, 12 bird species, 37 reptile species and six amphibian species, with porcupines being one of the species used.

The porcupine is widely used in traditional medicine for centuries. Local people in Betung Kerihun National Park, West Kalimantan, Indonesia used porcupine in their traditional medicine (Putra *et al*. 2008). In Kalimantan (Borneo), porcupine’s quills are grinded into flour to treat acne and burned to relieve back pain (Inayah 2016; Krisyanto *et al*. 2019). Azliza *et al*. (2012) reported that the people in Ulu Kuang Village, Malaysia used the porcupine’s quills to treat asthma and breathlessness. In Java, some locals use the porcupine’s quills as a medicine for toothaches and ulcers (Inayah 2016; Krisyanto *et al*. 2019). Gomez (2021) reported that the porcupine’s quills are used for traditional medicine in Aceh, Bali and Kalimantan (Borneo). Another part of porcupine, bezoar stone, was reported to be used in traditional medicine in South East Asia and Europe to treat cancer, poisoning, fever and typhoid (Heinrich *et al*. 2020). Khan *et al*. (2019), reported that the porcupine’s bezoar stone is scientifically proven to have anticancer activity through *in vivo* and *in vitro* studies. However, these reported traditional medicine (ethnomedicine) of porcupine need scientific support to reveal the potency as medicinal candidate since the study is still limited.

One species of the porcupine that is thought to be used in traditional medicine is the Sunda porcupine (*Hystrix javanica*, F.Cuvier, 1823), which is reported to be found in certain regions of Indonesia including Java, Bali, Sumbawa, Flores, Lombok, Madura and Tonahdjampea (Van Weers 1979, 1983; Woods & Kilpatrick 2005; Aplin 2016). Recent research has investigated the potential of the Sunda porcupine in animal-based medicine. Prawira *et al*. (2020) reported the rapid wound healing in this species, while Gifardi *et al*. (2022) demonstrated that Sunda porcupine’s quills hexane extract could inhibit the growth of *Staphylococcus aureus*, a bacteria that infect the skin at certain concentration levels. Furthermore, Anita *et al*. (2017) reported that the tail meat of Sunda porcupine possesses aphrodisiac potency.

The exploration of active compounds from Sunda porcupine’s quills is of interest since its utilisation in traditional medicine and the lack of study in this area. This study aims to identify active compounds in Sunda porcupine’s quills ethanolic crude extract as well as to evaluate its antioxidant and antimicrobial properties. This research is expected to provide new knowledge and contribute to the discovery of traditional medicine as a potential source of drugs.

## MATERIALS AND METHODS

### Sample Extraction

Sunda porcupine’s quills were collected from the remains of physiological research samples and the simplisia were obtained by drying the quills in an oven (Isuzu model AT-S13, Japan) at 50°C for five days. The simplisia were grounded to a size of 60 mesh and had a water content of 9.1%. A maceration method was used to extract the active compound inside simplisia with a simplisia to solvent ratio of 1:30 using 70% ethanol (Merck, Germany). The crude extract was obtained by evaporating all solvent using a rotary evaporator (IKA model RV 10D S99, Germany) at 50°C. Then, it was stored at 4°C until further use (Budiman *et al*. 2021).

### Determining Antioxidant Activity using DPPH-free Radicals Scavenging Assay

The 1,1-diphenyl-2-picrylhydrazyl (DPPH) (Sigma Aldrich, Germany) free radical was used to measure the antioxidant activity of the Sunda porcupine’s quills crude extract. The procedure used was adapted from Handayani *et al*. (2022) and Aryal *et al*. (2019) with slight modifications. The sample was dissolved in methanol (Merck, Germany) at concentrations ranging from 0 μg/mL to 250 μg/mL. A mixture of 2 mL of the sample and 2 mL of 0.1 mM DPPH was incubated in the dark for 30 min. The absorbance of the sample was measured using a UV-Vis spectrophotometer (Thermo Fisher Scientific model Genesys 10-S, US) at a wavelength of 517 nm. The antioxidant activity was calculated using the following formula:


$$\text{Antioxidant activity }(\%)=\frac{\text{Absorbance blank}-\text{Absorbance sample}}{\text{Absorbance blank}}\times 100\%$$

To determine the inhibitory concentration of 50% (IC_50_) value, the antioxidant activity score obtained from the DPPH assay was plotted against the concentration of the sample. The concentration of the sample that caused a 50% reduction of DPPH was determined from the graph as the IC_50_ value. Sample with lower IC_50_ value were considered more effective in neutralising free radicals.

### Determining Total Phenolic Content (TPC)

A sample was first dissolved in distilled water to obtain a 1,000 μg/mL solution. A standard curve was created using gallic acid with a range of serial concentrations from 0 μg/mL to 200 μg/mL. A 0.2 mL sample or standard was added into 1.8 mL of distilled water and 0.2 mL of Folin-Ciocalteu reagent (Merck, Germany). The solution was homogenised and incubated for 6 min. After that, 2 mL of Na_2_CO_3_ 7% (w/v) (Merck, Germany) was added to the solution, homogenised and incubated for 90 min. The absorbance was then measured at 750 nm using a UV-Vis spectrophotometer. TPC was calculated in milligram gallic acid equivalent per gram (mgGAE/g) sample (Maeng *et al*. 2017).

### Determining Total Flavonoid Content (TFC)

The determination of TFC followed the Dowd method as described by Aryal *et al*. (2019). A sample solution of 1,000 μg/mL was prepared using distilled water. Quercetin (Sigma Aldrich, China) was used as a standard with serial concentrations ranging 0 μg/mL to 100 μg/mL. A 1 mL of prepared sample or standard was added to a mixture of 0.2 mL AlCl_3_ 10% (w/v) (Merck, Germany) in methanol (Merck, Germany), 0.2 mL CH_3_COOK 1 M (Merck, Germany), and 5.6 mL distilled water. The solution was homogenised and incubated for 30 min. The absorbance was measured with a UV-Vis spectrophotometer at 415 nm. TFC was calculated in milligram quercetin equivalent per gram (mgQE/g) sample.

### Determining Antimicrobial Activity using Resazurin Assay

The antimicrobial activity was assessed using microdilution method incorporated with resazurin (Sigma Aldrich, China) as the indicator of cell viability (EUCAST 2022; Sarker *et al*. 2007). The assay was conducted in 96-well plate against *Escherichia coli* (*E. coli*), *Staphylococcus aureus* (*S. aureus*), *Bacillus subtilis* (*B. subtilis*), *Pseudomonas aeruginosa* (*P. aeruginosa*) and *Candida albicans* (*C. albicans*). Prior to antimicrobial assay, the target microbes were grown in Luria Bertani (LB) broth (Himedia, India) and incubated overnight in an incubator


$$\%\;\text{Inhibition}=\left(1-\left(\frac{\text{Fluorescence sample}-\text{Fluorescence of control }(-)}{\text{Fluorescence of control }(+)-\text{Fluorescence of control }(-)}\right)\right)\times 100\%$$

shaker (Bio-Shaker BR-300LF, Japan). The target microbes were then adjusted using McFarland turbidity standard 0.5 (Himedia, India) and diluted 1,000×, so that the final concentration of the cells was ± 1.5 × 105 CFU/mL. Antimicrobial assay of the extract was then conducted with concentration 100 mg/mL as the starting point and serially diluted to several concentrations followed by overnight incubation at 37°C. After the addition of 30 μL 0.1% resazurin, the cell suspension then incubated overnight and read under fluorescence with multimode reader Varioskan Lux (Thermo Fisher Scientific, US) with 530 excitation and 590 emission. The fluorescence data were used to determine the inhibition activity (%) using the following equation:

The IC_50_ was determined through a dose-response relationship using linear regression analysis, with the transformation of the concentration to a logarithmic scale.

### Identification of Active Compound Using GCMS

The Sunda porcupine’s quills ethanolic crude extract was dissolved with dichloromethane (Merck, Germany) to make a 1,000 μg/mL solution. It was then filtered using a minisart syringe membrane 0.22 μm (Sartorius, Germany). The filtrate was injected into a gas chromatography-mass spectrophotometer (GCMS) instrument (Shimadzu GCMS-QP 2010 Ultra, Japan) equipped with an Rtx-5MS column (5% diphenyl: 95% dimethyl-polysiloxane) with length of 30 m and diameter of 0.25 mm. The mobile phase consisted of ultra-high purity helium on 30 kPa. The injector temperature was set at 200°C, the ion source at 230°C, and the interphase at 280°C, with a splitless injection mode. The oven temperature program was initiated at 60°C and increased to 150°C at a rate of 10°C/min and held for 3 min. The resulting chromatogram and m/z were compared with the National Institute of Standards and Technology (NIST-11) database to identify the active compound.

## RESULTS

### Antioxidant Activity, TPC and TFC

**Figure 1:**
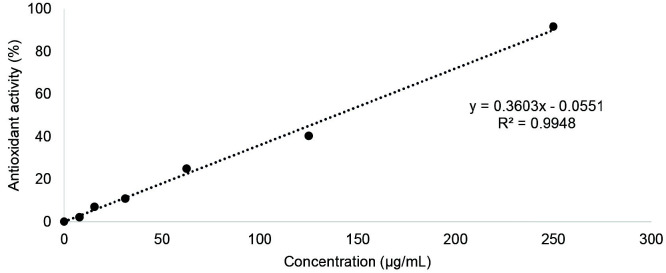
Antioxidant activity of Sunda porcupine’s ethanolic crude extract using DPPH free radical scavenging assay.

The antioxidant activity of Sunda porcupine’s ethanolic crude extract was assessed at varying concentrations, ranging from 0 μg/mL to 250 μg/mL. The curve of antioxidant activity against the sample concentration was determined with a regression equation of y = 0.3603× – 0.0551 and R^2^ = 99.48% as illustrated in Fig. 1. The IC_50_ of the extract’s antioxidant against DPPH free radical scavenging activity was determined to be 138.93 μg/mL.

**Figure 2:**
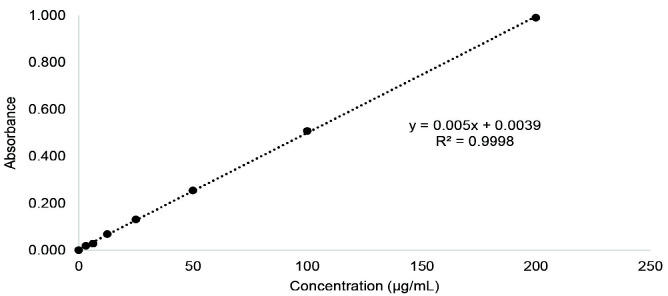

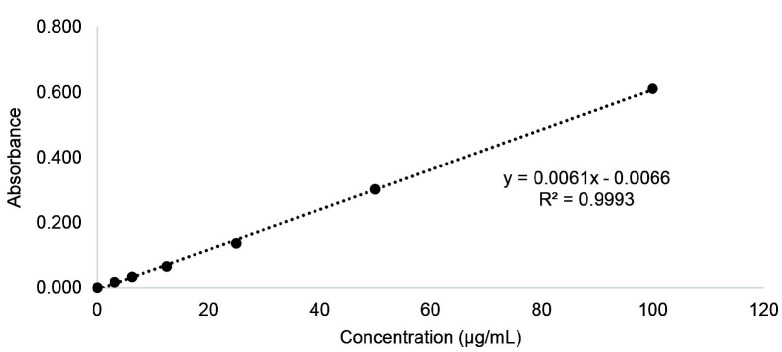
Standard curve of (a) gallic acid and (b) quercetin.

The standard curve of gallic acid and quercetin were used to determine the TPC and TFC as illustrated in Fig. 2. The linear regression equation of gallic acid and quercetin were derived as y = 0.005× + 0.0039 (R^2^ = 99.98%) and y = 0.0061× – 0.0066 (R^2^ = 99.93%), respectively. The TPC and TFC of the extract were calculated to be 27.29 ± 2.20 mgGAE/g sample and 27.09 ± 1.66 mgQE/g sample, respectively.

### Antimicrobial Activity

**Figure 3:**
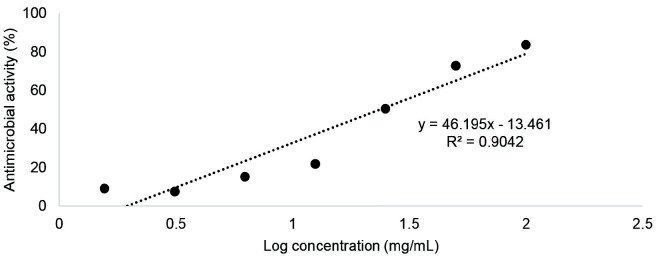

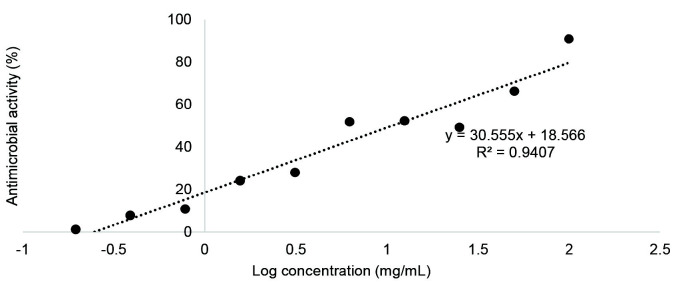

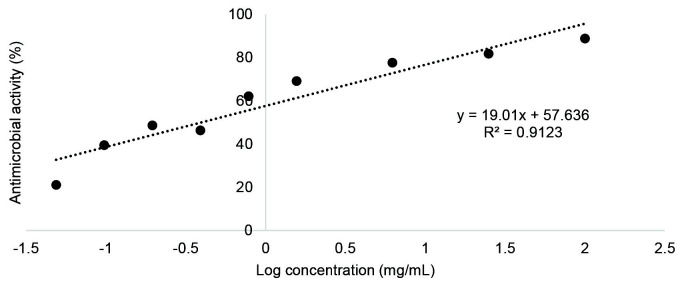

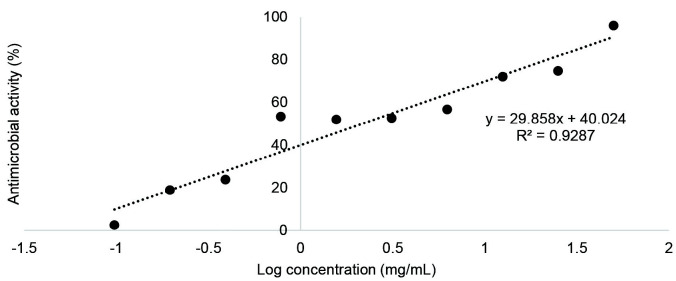

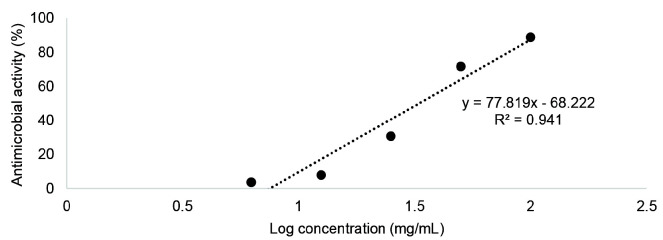
Antimicrobial activity of Sunda porcupine’s quills ethanolic crude extract against (a) *E. coli*, (b) *P. aeruginosa*, (c) *S. aureus*, (d) *B. subtilis*, and (e) *C. albicans*.

In the present study, the antimicrobial activity of Sunda porcupine’s quills ethanolic crude extract was evaluated for its antimicrobial activity against various microorganisms, including *E. coli*, *P. aeruginosa*, *S. aureus*, *B. subtilis* and *C. albicans*. The result is presented in Fig. 3, which shows the linear regression of antimicrobial activity against the log concentration of the extract for each microorganism.

The data revealed that the extract possessed significant antimicrobial activity against all the tested microorganisms. The highest antimicrobial activity was observed against *S. aureus* with an IC_50_ of 0.40 mg/mL (Table 1), indicating that the extract could be a potential source of antibacterial agents, mainly to Gram positive bacteria.

The linear regression equation for *E. coli*, *P. aeruginosa*, *S. aureus*, *B. subtilis* and *C. albicans* were y = 46.195× – 13.461 (R^2^ = 90.42%), y = 30.555× + 18.566 (R^2^ = 94.07%), y = 19.01× + 57.636 (R^2^ = 91.23%), y = 29.858× + 40.024 (R^2^ = 92.87%), and y = 77.819× + 68.222 (R^2^ = 94.10%) respectively with y-axis representing the percentage of antimicrobial activity and x-axis representing the log concentration of the extract (Fig. 3). These findings suggest that Sunda porcupine’s ethanolic crude extract could be a promising candidate for the development of novel antimicrobial agents.

### Identification of Active Compound Using GCMS

**Figure 4:**
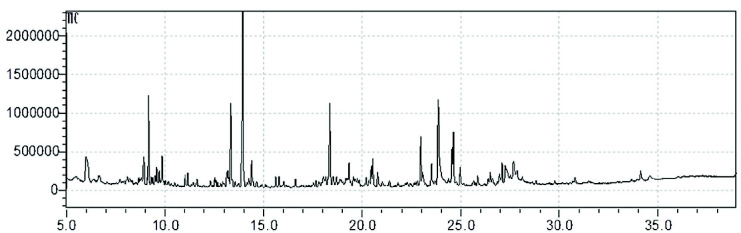
Chromatogram of Sunda porcupine’s quills ethanolic crude extract.

The GCMS analysis of the extract resulted in the chromatogram as shown in Fig. 4. After comparing the m/z data with the NIST-11 database, a total of 24 compounds were identified and are listed in Table 2. Among these compounds, six exhibit a relatively high intensity proportional to the percentage of area (more than 5%), including butylated hydroxytoluene (20.94%), L-(+)-ascorbic acid 2,6-dihexadecanoate (14.60%), eicosane (8.86%), 5-methyl-1-phenylbicyclo [3.2.0] heptane (8.21%), pentadecane (6.88%), and hexadecane (6.30%). The highest intensity to the percentage of area in chromatogram was found at retention time of 13.952 min and presumably represent butylated hydroxytoluene. It has been reported by Ayaz *et al*. (1980) and Lim *et al*. (1987) that it possesses antioxidant and antimicrobial properties. Furthermore, the second highest intensity was found at retention time of 23.860 min which presumably represent L-(+)-ascorbic acid 2,6-dihexadecanoate, also known as an antioxidant and antimicrobial agent (Igwe & Okwunodulu 2014; Hadi *et al*. 2016).

## DISCUSSION

The Sunda porcupine, an endemic mammal species of Indonesia, has a relatively wide distribution across several regions of the country, including Java, Bali, Sumbawa, Flores, Lombok, Madura and Tonahdjampea (Van Weers 1979, 1983; Woods & Kilpatrick 2005; Aplin 2016). This broad distribution has fostered a connection between the Sunda porcupine and the local people resulting traditional knowledge, including ethnobiology and ethnomedicine. This traditional knowledge is frequently not adequately documented and is instead passed down orally from generation to generation, leading to difficulties in accessing this information. Some indigenous communities in Indonesia are reported to use the Sunda porcupine’s quills for medicinal purposes such as treating acne, relieving backpain, curing ulcer and relieving toothache (Inayah 2016; Krisyanto *et al*. 2019). Furthermore, the Sunda porcupine’s quills is a unique skin derivate that provides an additional protective layer against harsh environment condition and acts as a defence tool against predators (Prawira *et al*. 2018). Therefore, it is plausible that the quills contain a certain compound that may be effective in combating environmental stress. The Sunda porcupine is a potential candidate from animal which can be used as medicinal sources since has filtered by its ethnomedicine from enormous natural resources. On the other hand, the use of Sunda porcupine is sustainable because the animal can reproduce easily. Female Sunda porcupine can breed twice a year which can give birth up to four young porcupines in one birthing period (Farida *et al*. 2019; Suyanto 2012). The Sunda porcupine was also reported to be successfully breed in captivity (Suyanto 2012). In captivity, the porcupine’s quills are a side-product since they shed periodically from the porcupine’s body. Therefore, the Sunda porcupine’s quills are sustainable for medicinal purposes because the porcupine is easily reproduced and the quills are shed periodically as a by-product in captivity.

The present study investigates the active compounds found inside the Sunda porcupine’s quills, specifically focusing on their antioxidant and antimicrobial properties. The quills were extracted using a 70% ethanol solvent via the maceration method. Ethanol was chosen as the solvent due to its safety profile in comparison to other organic solvents. The maceration process was selected to minimise the risk of damaging the active compounds through the application of heat. The extract in this study was identified using GCMS which successfully identified 24 active compounds as shown in Table 2. Most of these compounds were identified as biologically active, which aligns with previous studies.

Antioxidants are compounds that can help reduce oxidative stress, mainly grouped as endogenous and exogenous antioxidant. The endogenous antioxidants are antioxidant normally produced by human’s body such as reduced glutathione, catalase, superoxide dismutase, glutathione peroxidase, and reductase. The exogenous antioxidants are usually taken from the environment by foods or supplements including some vitamins like vitamins A, C and E (Adwas *et al*. 2019). The antioxidants work by scavenging or stimulating the breakdown of free radicals, both forms of antioxidants can help prevent the production of the free radicals (Maroof & Gan 2022). The antioxidant activity of Sunda porcupine’s quills ethanolic crude extract was determined using DPPH free radicals scavenging assay in various concentrations. Furthermore, the antioxidant activities with their respective concentrations were plotted in a linear regression to determine the antioxidant IC_50_. The antioxidant IC_50_ of the extract in present study was 138.93 μg/mL, indicating the concentration of the extract required to neutralise 50% of free radicals. A lower IC_50_ value indicates a smaller concentration of the sample needed to neutralise free radicals. The antioxidant properties are mostly caused by the content of phenolic and flavonoid compounds (Aryal *et al*. 2019; Maeng *et al*. 2017). The TPC and TFC of the extract was 27.29 ± 2.20 mgGAE/g and 27.09 ± 1.66 mgQE/g, respectively. The flavonoids are a part of phenolics. The score of TPC and TFC are close, indicating the phenolics content are mostly in the form of flavonoids.

Moreover, the antioxidant properties of the extract were in line with the identified compound obtained from GCMS analysis. There are five compounds with a total of 47.33%, proportional to the percentage of area in chromatogram responsible with antioxidant properties. These are butylated hydroxytoluene (20.94%), L-(+)-ascorbic acid 2,6-dihexadecanoate (14.60%), eicosane (8.86%), methyl ester hexadecanoic acid (1.70%), and 1-iodo-hexadecane (1.23%). Butylated hydroxytoluene was reported as an antioxidant to inhibit free radicals production for medicine and cosmetics (Ershov & Volod’kin 1962). The L-(+)-ascorbic acid 2,6-dihexadecanoateis a vitamin C derivative and it is important as a lipophilic antioxidant, antitumour, wound healing and antimicrobial properties (Igwe & Okwunodulu 2014; Hadi *et al*. 2016). Eicosane is a monoterpenic hydrocarbon and is reported to have antioxidant and anti-inflammatory properties by inhibiting the release of cytokines such as histamine, bradykinin, prostaglandins, thromboxanes and leukotrienes in rats (Kazemi 2015; Okechukwu 2020). Methyl ester hexadecanoic acid (methyl palmitate) is a fatty acid group with antioxidant, hypocholesterolaemia, and antiandrogenic properties (Hema *et al*. 2011; Astiti & Ramona 2021). The 1-iodo-hexadecane has been reported in some plant extract and possesses antioxidant properties (Kim *et al*. 2022).

The present study also investigates the antimicrobial properties of Sunda porcupine’s quills ethanolic crude extract, in addition to its antioxidant properties. Resazurin assay was used in this study to determine the antimicrobial activity of the extract against *E. coli*, *P. aeruginosa*, *S. aureus*, *B. subtilis* and *C. albicans*. The use of resazurin microdilution assay was selected since it can provide more accurate result through spectrophotometry, allowing for precise analysis signal readings. The antimicrobial activity was determined by measuring the resazurin readings after 24 h of incubation for bacteria and 72 h of incubation for yeast. Resazurin (7-hydroxy-3H-phenoxazin-3-one 10-oxide) is a blue dye that can be irreversibly reduced by oxidoreductase in active bacteria to a pink and highly red fluorescent substance called resorufin (Chakansin *et al*. 2022). This method is based on detection of microbial viability by observing the colour change of resazurin from blue to purple or pink (Jia *et al*. 2020). The test was considered positive if the well contents were blue in colour, indicating the extract inhibits the growth of microbial, and negative if the well contents were pink, indicating the microbial is still growing in the medium wells. The test results were valid if negative control wells (without extract) remained pink (Germ *et al*. 2019).

The antimicrobial IC_50_ values of Sunda porcupine quills extract against *E. coli*, *P. aeruginosa*, *S. aureus*, *B. subtilis* and *C albicans* was 23.65 mg/mL, 10.68 mg/mL, 0.40 mg/mL, 2.16 mg/mL and 33.05 mg/mL, respectively. These results indicate that the Sunda porcupine quills extract has a broad antimicrobial range as it can inhibit the growth of Gram-positive bacteria, Gram-negative bacteria, spore-forming bacteria and yeast. In particular, the *S. aureus* bacteria species was highly sensitive for antimicrobial activity. However, the antimicrobial assay was limited to several species in present study. The further research related to the antimicrobial assay with other microbial species may complete the potential of Sunda porcupine’s quills as an antimicrobial agent. Sunda porcupine’s quills ethanolic crude extract could inhibit the activity of *S. aureus* at the smallest concentration (0.4 mg/mL or 0.04% equivalent). The concentration of the Sunda porcupine’s quills ethanolic crude extract required to inhibit *S. aureus* in the present study was found to be significantly lower (0.04%) than reported by Gifardi *et al*. (2022) for the Sunda porcupine’s quills extracted using hexane solvent, which required at least a concentration of 25% for the inhibition of *S. aureus* growth. These findings suggest that the antimicrobial compounds in Sunda porcupine’s quills are more soluble in polar solvent such as ethanol 70%. Further research on the extraction and identification of the active compound in Sunda porcupine’s quills could lead to the development of novel and effective antimicrobial agents.

Furthermore, the antimicrobial properties of the Sunda porcupine ethanolic crude extract also appropriate with the GCMS analysis. As much as 11 identified compounds have been reported to possess antimicrobial properties. In total, it is about 70.12% of identified compound, proportional to the percentage of area in chromatogram, that possess antimicrobial properties. These compounds include butylated hydroxytoluene (20.94%), L-(+)-ascorbic acid 2,6-dihexadecanoate (14.60%), eicosane (8.86%), pentadecane (6.88%), hexadecane (6.30%), octadecanoic acid (3.48%), tetradecane (3.07%), benzenepropanoic acid, 3,5-bis(1,1-dimethylethyl)-4-hydroxy-, methyl ester (1.76%), methyl ester hexadecanoic acid (1.70%), 2-isopropyl-5-methyl-1-heptanol (1.30%) and 1-iodo-hexadecane (1.23%).

Butylated hydroxytoluene have been reported as antimicrobial to inhibit growth of some microorganism (Ayaz *et al*. 1980; Lim *et al*. 1987). The L-(+)-ascorbic acid 2,6-dihexadecanoate is an ascorbic acid derivate which is potential to prevent and treat common cold, gum diseases, acne, skin infection, tuberculosis, dysentery, and dental caries (Igwe & Okwunodulu 2014). Eicosane have been reported as antifungal, antibacterial, antitumour, inhibit foodborne pathogen, and has cytotoxic effect (Hsouna *et al*. 2011; Yogeswari *et al*. 2012; Akpuaka *et al*. 2013; Kazemi 2015; Okechukwu 2020). Pentadecane is reported as an antimicrobial compound by inhibited growth of *E. coli* and *S. typhi* that involves interaction with a cell’s electrical channel protein in silico analysis. Channel protein plays a role in pumping protons concerning the metabolism of amino acids of *E. coli* and *S. typhi* (Firdaus *et al*. 2019; Martinac *et al*. 1987). Octadecanoic acid or stearic acid is a fatty acid that displays antibacterial activity towards Gram-positive and Gram-negative bacteria (Abdalaziz *et al*. 2017; Casillas-Vargas *et al*. 2021). Hexadecane, tetradecane, 2-isopropyl-5-methyl-1-heptanol and 1-iodo-hexadecane has reported to possess antifungal and antimicrobial properties (Selvin *et al*. 2009; Yogeswari *et al*. 2012; Akpuaka *et al*. 2013; Kim *et al*. 2022). Benzenepropanoic acid was also reported to be effective against microbes such as *E. coli*, *K. pneumoniae*, *P. aeruginosa* and *C. albicans* (Amrati *et al*. 2021). Benzoic acid alone is known as a nonspecific antimicrobial agent with a wide spectrum of the activities against human pathogenic fungi and bacteria (Innocenti *et al*. 2009; Krátký *et al*. 2012). Methyl ester hexadecanoic acid was reported as antibacterial by disrupting bacterial cell wall and cell membrane (Astiti & Ramona 2021).

## CONCLUSION

As much as 24 active compounds were identified from Sunda porcupine’s quills ethanolic crude extract using GCMS. The extract was investigated and showed antioxidant and antimicrobial properties. The IC_50_ of antioxidant was 138.93 μg/mL, while the IC_50_ of antimicrobial against *E. coli*, *P aeruginosa*, *S. aureus*, *B. subtilis* and *C. albicans* were 23.65 mg/mL, 10.68 mg/mL, 0.40 mg/mL, 2.16 mg/mL and 33.05 mg/mL, respectively. The antioxidant properties were also investigated through the determination of TPC and TFC with values of 27.29 ± 2.20 mgGAE/g and 27.09 ± 1.66 mgQE/g, respectively. There were five identified compounds thar serve as antioxidant and 11 identified compounds that serve as antimicrobial. The two highest intensities to the percentage of area in chromatogram were butylated hydroxytoluene (20.94% with RT = 13.952 min) and L-(+)-ascorbic acid 2,6-dihexadecanoate (14.60% with RT = 23.860 min). Both these compounds have been reported as antioxidant and antimicrobial agents. This study provides scientific validation for the use of the Sunda porcupine’s quills in traditional medicine and highlights the potential for further research in animal bioprospecting.

## Figures and Tables

**Table 1:** The antimicrobial IC_50_ of Sunda porcupine’s quills ethanolic crude extract.

Microorganism	Group	IC_50_ (mg/mL)
*Escherichia coli*	Gram negative	23.65
*Pseudomonas aeruginosa*	Gram negative	10.68
*Staphylococcus aureus*	Gram positive	0.40
*Bacillus subtilis*	Gram positive sporadic	2.16
*Candida albicans*	Fungi	33.05

**Table 2:** The active compound identified by GCMS instrument with NIST-11 database.

No.	Retention time (min)	Area (%)	Name	Molecular formula	Similarity (%)	Role(s)
1	8.921	3.07	Tetradecane	C_14_H_30_	75	Antifungal and antibacterial (Ozdemir *et al*. 2004)
2	9.167	6.88	Pentadecane	C_15_H_32_	92	Antimicrobial (Martinac *et al*.1987; Firdaus *et al*. 2019)
3	9.570	1.30	2-Isopropyl-5-methyl-1-heptanol	C_11_H_24_O	87	Antimicrobial (Selvin *et al*. 2009)
4	9.700	1.04	2-methyl-1-decanol	C_11_H_24_O	87	–
5	9.855	2.22	2,6,11-trimethyl-dodecane	C_15_H_32_	91	–
6	11.024	1.08	E-14-Hexadecenal	C_16_H_30_O	84	–
7	13.199	1.33	2,6,11,15-tetramethyl-hexadecane	C_20_H_42_	82	Flavouring agent (Pammi *et al*. 2021)
8	13.346	6.30	Hexadecane	C_16_H_34_	90	Antifungal and antibacterial (Yogeswari *et al*. 2012; Akpuaka *et al*. 2013)
9	13.952	20.94	Butylated hydroxytoluene	C_15_H_24_O	95	Antimicrobial (Ayaz *et al*. 1980; Lim *et al*. 1987)
10	18.020	1.15	Dodecyl ester chloroacetic acid	C_14_H_27_ClO_2_	88	–
11	18.361	8.86	Eicosane	C_20_H_42_	87	Antifungal, antibacterial, inhibit foodborne pathogen, antitumour and cytotoxic effect (Hsouna *et al*. 2011; Yogeswari *et al*. 2012; Akpuaka *et al*. 2013; Kazemi 2015; Okechukwu 2020)
12	18.538	1.23	1-iodo-hexadecane	C_16_H_33_I	75	Inhibitory effect on AD-like lesions, antimicrobial, antioxidant and anticancer (Kim *et al*. 2022)
13	19.167	1.07	2-methoxycarbonyl-2-methylbrendane	C_12_H_18_O_2_	65	–
14	20.459	1.73	Benzoic acid, 2-fluoro-5,6-dimethoxy	C_9_H_9_FO_4_	59	–
15	20.539	2.70	(E)-2-Heptenedioic acid, 4-cyclopropyl-, dimethyl ester	C_12_H_18_O_4_	53	–
16	20.787	1.54	Hexadecanal	C_16_H_32_O	93	–
17	22.967	4.86	Heptadecane, 2-methyl	C_18_H_38_	89	–
18	23.055	1.70	Methyl ester hexadecanoic acid	C_17_H_34_O_2_	92	Antioxidant, antifungal and antimicrobial (Hema *et al*. 2011; Astiti & Ramona 2021)
19	23.509	1.76	Benzenepropanoic acid, 3,5-bis(1,1-dimethylethyl)-4-hydroxy-, methyl ester	C_18_H_28_O_3_	91	Antimicrobial (Ahmad *et al*. 2012; Akpuaka *et al*. 2013; Amrati *et al*. 2021)
20	23.860	14.60	L-(+)-ascorbic acid 2,6-dihexadecanoate	C_38_H_68_O_8_	86	Antioxidant, antitumour, wound healing and antimicrobial properties (Igwe & Okwunodulu 2014; Hadi *et al*. 2016)
21	24.550	3.51	5-methyl-1-phenylbicyclo[3.2.0] heptane	C_14_H_18_	69	Antivirus (Poongulali & Sundararaman 2016)
22	24.959	1.63	Oxirane, heptadecyl	C_19_H_38_O	93	–
23	27.667	3.48	Octadecanoic acid	C_18_H_36_O_2_	83	Antibacterial and antifungal (Akpuaka *et al*. 2013)

24	27.845	1.32	Tetracosane	C_24_H_50_	87	Anticancer (Paudel *et al*. 2019)

*Note:* (–) Unknown roles
